# Reactive oxygen species are required for driving efficient and sustained aerobic glycolysis during CD4^+^ T cell activation

**DOI:** 10.1371/journal.pone.0175549

**Published:** 2017-04-20

**Authors:** Dana M. Previte, Erin C. O’Connor, Elizabeth A. Novak, Christina P. Martins, Kevin P. Mollen, Jon D. Piganelli

**Affiliations:** 1 Department of Surgery, Children’s Hospital of Pittsburgh, University of Pittsburgh Medical Center, Pittsburgh, Pennsylvania, United States of America; 2 Department of Immunology, School of Medicine, University of Pittsburgh, Pittsburgh, Pennsylvania, United States of America; University of South Alabama, UNITED STATES

## Abstract

The immune system is necessary for protecting against various pathogens. However, under certain circumstances, self-reactive immune cells can drive autoimmunity, like that exhibited in type 1 diabetes (T1D). CD4^+^ T cells are major contributors to the immunopathology in T1D, and in order to drive optimal T cell activation, third signal reactive oxygen species (ROS) must be present. However, the role ROS play in mediating this process remains to be further understood. Recently, cellular metabolic programs have been shown to dictate the function and fate of immune cells, including CD4^+^ T cells. During activation, CD4^+^ T cells must transition metabolically from oxidative phosphorylation to aerobic glycolysis to support proliferation and effector function. As ROS are capable of modulating cellular metabolism in other models, we sought to understand if blocking ROS also regulates CD4^+^ T cell activation and effector function by modulating T cell metabolism. To do so, we utilized an ROS scavenging and potent antioxidant manganese metalloporphyrin (MnP). Our results demonstrate that redox modulation during activation regulates the mTOR/AMPK axis by maintaining AMPK activation, resulting in diminished mTOR activation and reduced transition to aerobic glycolysis in diabetogenic splenocytes. These results correlated with decreased Myc and Glut1 upregulation, reduced glucose uptake, and diminished lactate production. In an adoptive transfer model of T1D, animals treated with MnP demonstrated delayed diabetes progression, concurrent with reduced CD4^+^ T cell activation. Our results demonstrate that ROS are required for driving and sustaining T cell activation-induced metabolic reprogramming, and further support ROS as a target to minimize aberrant immune responses in autoimmunity.

## Introduction

Type 1 diabetes (T1D) is an autoimmune disease where self-reactive T cells escape into the periphery and target pancreatic β cells for destruction. While T1D progression results from the interplay between various immune cell types, CD4^+^ T cells are considered the principal contributor to disease pathology [1, 2]. We and others have demonstrated that reactive oxygen species (ROS) play an important role in driving the immunopathology exhibited in T1D [3, 4]. Antigen presenting cells (APCs), like macrophages [5], and CD4^+^ T cells [6] express functional NADPH oxidases (NOX) which generate ROS upon APC-induced T cell activation. Both NOX [7] and mitochondrial-derived ROS from the T cell itself [8] are necessary for optimal CD4^+^ T cell activation. These ROS, with cytokines, serve as the third signal, during T cell activation. In combination with T cell receptor (TCR; signal 1) and co-stimulatory molecule (signal 2) engagement, these three signals enable cell cycle entry [9] and effector function acquisition [7].

Recently, interest has grown in understanding the role of cellular metabolism in fulfilling the objectives of T cell activation and effector function. Under homeostatic conditions, naïve CD4^+^ T cells remain relatively quiescent and rely predominantly on oxidative phosphorylation (OXPHOS) to meet basal metabolic needs [10]. Upon antigen (e.g. β cell-derived antigens in T1D) encounter, naïve CD4^+^ T cells become activated and have two main goals–to clonally expand and to differentiate into effector T cells. To meet these goals during activation, CD4^+^ T cells undergo dynamic metabolic reprogramming by transitioning to aerobic glycolysis [10–13], also known as the Warburg Effect, which was first characterized in tumors [12, 14]. The utilization of aerobic glycolysis by activated CD4^+^ T cells supports increased macromolecule biosynthesis, aiding in daughter cell formation and effector molecule production, along with more rapid production of ATP as compared to OXPHOS [10–12].

In both tumors and T cells, Myc is a predominant player in coordinating increased glycolysis and cell proliferation [14–17]. Upstream, activation of mammalian target of rapamycin (mTOR) signaling is critical for Myc expression and thus aerobic glycolysis, as treatment with the mTOR inhibitor rapamycin results in dampened lactate production, proliferation, and cytokine production in CD4^+^ T cells [18, 19]. In contrast, AMP-activated protein kinase (AMPK) is a known inhibitor of mTOR and is responsible for enhancing oxidative metabolism to restore the ATP to AMP ratio [20, 21]. Overexpression of AMPK in tumors inhibits the Warburg Effect, whereby tumors demonstrate reduced size and lactate production [22]. Similarly, AMPK activation in T cells results in reduced mTOR activation, diminished effector differentiation, and hyporesponsiveness [23]. These results highlight that the interplay between mTOR and AMPK strongly dictates T cell metabolic and functional outcome.

Highly proliferative cells in various models demonstrate enhanced aerobic glycolysis, indicating its requirement for sustaining rapid division. Targeting tumor metabolism via the use of glycolytic inhibitors like 2-deoxyglucose, have proven to be effective in reducing tumor burden and metastasis [24]. The efficacy of metabolic modulation in cancer, and the metabolic similarities between proliferating tumor cells and effector CD4^+^ T cells, indicate a potential avenue for controlling aberrant T cell responses (like those in autoimmunity) by targeting T cell metabolism. Indeed, others have demonstrated potential for ameliorating autoimmunity by metabolic manipulation [25, 26]; however, there remains a large gap in understanding the mechanisms by which specifically T cell metabolism is controlled.

Additionally, many metabolic regulators demonstrate redox sensitivity, including the transcription factors HIF-1α [24] and NF-κB [27], and AMPK [28], to name a few, underscoring the potential for redox regulation in modulating metabolism. We and others have shown that a manganese metalloporphyrin, Mn(III) meso tetrakis (N -alkylpyiridinium-2-yl) porphyrin, or MnP, is capable of scavenging ROS (i.e. hydrogen peroxide and superoxide) [29, 30], inhibiting lipid peroxidation [31], and performing redox reactions in cellular systems [32, 33]. As T1D is known to be driven by increased oxidative stress [3, 34], our laboratory has demonstrated that inhibition of ROS during immune activation results in dampened CD4^+^ T cell responses, thus inhibiting T1D progression [35–38]. Specifically, work by *Delmastro-Greewood et al*. showed that treating NOD.BDC.2.5.TCR-Tg mice with MnP *in vivo* for 7 days resulted in increased glucose oxidation and aconitase activity in naïve splenocytes, indicative of enhanced OX PHOS, the predominant pathway used by naïve immune cells [39]. While these studies did demonstrate metabolic alterations due to MnP treatment, they were conducted using naïve immune cells that had no prior exposure to their cognate antigen. As previously stated, T cell metabolic reprogramming occurs only during antigen-mediated activation; therefore, we sought to expand our understanding of the role of ROS and metabolism during such activation events.

Based on these previous studies, we hypothesized that redox modulation by MnP during CD4^+^ T cell activation would inhibit the transition to aerobic glycolysis, and thus, minimize proliferation and effector function. Our data demonstrate that MnP treatment resulted in reduced Myc upregulation, glycolytic enzyme expression, and lactate production, collectively indicating inhibition of aerobic glycolysis. These results were in part due to diminished mTOR signaling. Interestingly, redox modulation enhanced activation of the mTOR inhibitor, AMPK, due to MnP’s high antioxidant activity. These data show that redox modulation inhibits the metabolic transition of CD4^+^ T cells by maintaining active AMPK and thus resulting in reduced mTOR signaling and Myc expression. These findings support that ROS are required during the transition from OX PHOS to aerobic glycolysis during T cell activation, and that disruption of ROS may serve as a viable target for modulating immune cell bioenergetics in autoimmune diseases like T1D.

## Materials and methods

### Animals

Non-obese diabetic (NOD), NOD.BDC2.5.TCR.Tg, and NOD.*scid* mice were maintained in the Rangos Research Center animal facility of the Children’s Hospital of Pittsburgh. Animal experiments were approved by the Institutional Animal Care and Use Committee (IACUC) of Children’s Hospital of Pittsburgh (Assurance Number A3187-01) and were in compliance with the laws of the United States of America. NOD.BDC2.5.TCR.Tg mice were sacrificed at 8–10 weeks of age for *in vitro* experiments. In this animal, all CD4^+^ T cells recognize epitopes formed by covalent cross-linking of proinsulin peptides and Chromogranin A (CHgA) in β cell secretory granules [40]. These T cells can be stimulated with a known peptide mimotope HRPI-RM that has been previously described [41], thus allowing us to examine the effects of MnP on an antigen-specific immune response physiologically relevant to T1D. NOD.*scid* animals, 6–8 weeks of age, were used for adoptive transfer experiments.

### Mn(III) meso tetrakis (N -alkylpyiridinium-2-yl) porphyrin

Mn(III) meso tetrakis (N -alkylpyiridinium-2-yl) porphyrin (MnP) was a generous gift from Dr. James Crapo, MD at National Jewish Health (Denver, CO). MnP was used at a concentration of 68 μM for *in vitro* experiments and a 10 mg/kg dose in all animal experiments.

### Splenocyte homogenization

NOD.BDC.2.5.TCR-Tg spleens were harvested and homogenized into single cell suspensions as previously described [35], and red blood cells were lysed using red blood cell lysis buffer (Sigma). CD4^+^ T cells were stimulated with their cognate peptide, mimotope (EKAHRPIWARMDAKK), at 0.05 μM, with or without MnP in complete splenocyte medium [7]. Splenocytes plated with media alone served as negative controls. Cells were collected for downstream analysis at 24–72 hours post-stimulation. Supernatants were collected for ELISA and lactate measurements.

### CD4^+^ T cell isolation and antibody stimulation

CD4^+^ T cells were isolated from whole NOD splenocytes by magnetic bead separation using mouse CD4 MicroBeads (Miltenyi) as per manufacturer’s instructions. Purity was assessed by flow cytometric staining pre- and post-isolation. For antibody stimulation, tissue culture plates were coated with αCD3 (0.5 μg/mL) and αCD28 (1.0 μg/mL) in phosphate buffered saline for 2 hours at 37°C, 5% CO_2_. The antibody solution was decanted and CD4^+^ T cells were plated at 5.0x10^5^ cells per well of a 96 well, flat-bottom plate, with or without 68 μM MnP. Unstimulated T cells served as negative controls.

### ROS production and cell viability

NOD or NOD.BDC.2.5 splenocytes were incubated in media alone or media with 68 μM MnP for 2 hours at 37°C. Cells were washed extensively in cold Hank’s Balanced Salt Solution (HBSS) and added to flow tubes at 1.0x10^6^ per tube. Dihydroethidium (DHE; Molecular Probes) or MitoSOX Red (Molecular probes) was diluted per manufacturer’s instructions, and cells were treated with a final concentration of 50 μM for 20 minutes (DHE) or 5 μM for 15 minutes (MitoSOX) at 37°C. PMA (500 ng/mL) and ionomycin (500 μg/mL) were added to the tubes and incubated at 37°C for indicated periods of time. Cells were read on an LSRII (BD Bioscience). DHE was read in the AmCyan channel using a 585/42 detector and 545LP filter [42], and MitoSox Red was detected in the PE channel. Mean Fluorescence Intensity (MFI) was determined using FlowJo Software (v10.1). Dye loaded, unstimulated control cells were used to determine background fluorescence, which was subtracted from stimulated values and graphed as change in MFI due to stimulation (delta MFI). Viability was assessed by 7AAD staining (BD Biosciences) as per manufacturer’s instructions. Surface staining for CD4 was performed prior to 7AAD staining. Viability was determined as the percentage of 7AAD negative cells.

### Protein lysates and Western blotting

Following stimulation, cells were harvested, washed with phosphate buffered saline (PBS), and sonicated in anti-pY lysis buffer (50 mM Tris pH 8.0, 137 mM NaCl, 10% glycerol, 1% NP-40, 1 mM NaF, 10 μg/ml leupeptin, 10 μg/ml aprotinin, 2 mM Na_3_VO_4_, and 1 mM PMSF). Protein concentration was determined by Bicinchoninic acid protein assay (Thermo Fisher Scientific). 25 μg of protein per sample were boiled in 6x Lammaeli buffer (BIORAD) for 5 minutes and separated SDS-PAGE gels. Samples were then transferred to PVDF membranes for 1–3 hours in 3% MeOH Tris-Glycine Transfer buffer (BIORAD). Western blots were blocked in 5% non-fat dry milk in Tris-buffered Saline with 1% Tween-20 (TBST). Blots were probed with the following antibodies in 5% BSA/TBST overnight at 4°C: Myc, pmTOR (Ser2448), total mTOR, p4E-BP1 (Thr70), pAMPK-α (Thr170), total AMPK, PFKFB3, p27 Kip1, and Cyclin D3 at 1:1000 (Cell Signaling), and Glut-1 (1:2000; Abcam). Blots were either probed with anti-rabbit secondary antibody (Cell Signaling; 1:2000) or goat anti-rabbit secondary antibody (Jackson Laboratories; 1:10,000) in 5% non-fat dry milk/TBST at RT for 1 hour. β-actin (Sigma) expression was used as a loading control. Protein expression was detected by chemilumenescence using ECL Plus reagent (Amersham Pharmacia Biotech) and the Fujifilm LAS-3000 Imaging system (FujiFilm Technologies). Multi Gauge software was used to process images (Fujifilm Life Science). Beta-actin expression served as a loading control.

### Flow cytometry for proliferation, cell cycle analysis, and glucose uptake

Cells were harvested following stimulation and incubated with Fc block (CD16/CD32; BD Biosciences) prior to staining for flow cytometry. Extracellular staining was performed at 4°C using CD4-APC or CD4-FITC (BD Biosciences) in FACS buffer (1% BSA in PBS). For cellular proliferation measurements, splenocytes were stained with 1 μM carboxyfluorescein succinimidyl ester (CFSE; Invitrogen) in PBS at 37°C for 15 minutes and isolated CD4^+^ T cells were labeled with Cell Proliferation Dye Violet (BD Bioscience) as per manufacturer’s instructions. Cells were extensively washed with PBS, plated for stimulation, and surface stained after harvest.

For cell cycle analysis, cells were fixed and permeabilized in 70% cold EtOH for 20 minutes on ice following stimulation and stored at 4°C until staining for flow analysis. Cells were washed with ice cold PBS two times to remove residual EtOH, and surfaced stained for CD4 as described above. After RNase treatment for 1 hour at 37°C, cells were incubated with propidium iodide (0.4 mg/mL; Invitrogen), and analyzed immediately. Media-treated splenocytes served as controls to set gates for no proliferation (CFSE) and cell cycle stages (PI).

To measure glucose uptake, stimulated cells were incubated with 100 μM 2-(*N*-(7-Nitrobenz-2-oxa-1,3-diazol-4-yl)Amino)-2-Deoxyglucose (2-NBDG; Molecular Probes), a fluorescent glucose analog (Life Technologies), for 10 minutes at 37°C prior to harvest [43]. Uptake was quenched with PBS. Cells were stained for surface CD4 expression and analyzed by flow cytometry live. Fluorescence was measured using a FACS Calibur or LSR II flow cytometer (BD Biosciences). All data were analyzed using FlowJo software (v10.1) and samples were gated on CD4^+^ cells.

### Cytokine and lactate measurements

Supernatants from cell cultures were analyzed for IFNγ and IL-2 by ELISA according to manufacturer’s instructions (BD Biosciences). ELISAs were read on a SpectraMax M2 microplate reader (Molecular Devices), and data were analyzed using SoftMax Pro version 5.4.2 software (Molecular Devices). Lactate, a byproduct of aerobic glycolysis, was measured in culture supernatants using the Accutrend Plus meter and lactate strips (Roche) [44, 45]. Samples with high concentrations of lactate were diluted 1:2 in dI H_2_0 to obtain a reading within the meter’s range.

### Gene expression as measured by quantitative Real-Time PCR (qRT-PCR)

At 24 hours post-stimulation *in vitro*, cells were harvested and washed extensively with PBS. 5.0 x 10^6^ cells were lysed using RLT buffer (Qiagen) and 25 gauge needles with 1 mL syringes. mRNA was isolated using the RNeasy kit (Qiagen) and concentration was determined using a NanoDrop 2000c spectrophotometer (Thermo Scientific). cDNA was synthesized from 0.5 μg mRNA using the RT^2^ First Strand Kit (Qiagen). Gene expression was quantified by qRT-PCR using the iQ SYBR Green Supermix (BIORAD) and iCycler (BIORAD). Murine glycolytic primer pair sequences were taken from *Wang et al*. [15]. *Ifnγ* primers were FWD 5’-AGGCCATCAGCAACAACATAAGCG-3’ and REV 5’- TGGGTTGTTGACCTCAAACTTGGC-3’. Cycling parameters were as follows: 5 min at 95°C, 30 s at 95°C, 30 s at 60°C, 30 s at 72°C (40 cycles of steps 2–4), 1 min at 95°C, and then samples were held at 4°C. Delta delta Ct values were normalized to expression of the control gene *rplo* (FWD 5’-GGCGACCTGGAAGTCCAACT-3’; REV 5’-CCATCAGCACCACAGCCTTC-3’) [46], in order to calculate relative expression. Mimotope and M + MnP expression values were normalized to those of unstimulated, media controls.

### Adoptive transfer model of T1D

Spleens from NOD.BDC.2.5.TCR.Tg animals were homogenized and processed as described above. Whole splenocytes were labeled with Cell Proliferation Violet (BD Biosciences) according to manufacturer’s instructions, and 1.0x10^7^ splenocytes were adoptively transferred into NOD.*scid* recipients i.v. One cohort of recipients was treated with 10 mg/kg MnP i.p. every day or s.c. every other day, starting the day prior to transfer. Serum was collected on days -1, 3, 7, 11, and 15 post-transfer to measure sLAG-3 by ELISA as an indication of T cell activation, as previously described [35]. T1D incidence was monitored by blood glucose post-transfer, and two consecutive readings of >350 mg/dL was deemed diabetic. At indicated time points, animals were sacrificed, and peripheral blood and spleens were taken for downstream analysis by flow cytometry. 1.0x10^6^ splenocytes were stained with surface antibodies for CD4, CD25, and LAG-3 following F_c_ receptor blockade (all from BD Bioscience). For intracellular pS6 staining of peripheral blood, red blood cells were lysed and then lymphocytes were surface stained. Following fixation and permeabilization using Cytofix/Cytoperm (BD Bioscience), cells were then stained using the pS6 Alexa 488 antibody (Cell Signaling). Cells were then analyzed by flow cytometry using a BD LSRII (BD Bioscience) and FlowJo software (v10.1).

### Statistical analysis

Data are given as mean values ± SEM, with n indicating the number of independent experiments or animals, unless otherwise indicated. Student’s t-test and Two-way ANOVA with Bonferroni post-hoc analysis were used where appropriate. Kaplan-Meier analysis was used to measure significance of diabetes incidence. A p-value of p < 0.05 was considered significant for all statistical analyses.

## Results

### Treatment of T cells with MnP effectively scavenges NADPH oxidase and mitochondrial-derived ROS and without toxicity

**Fig 1:**
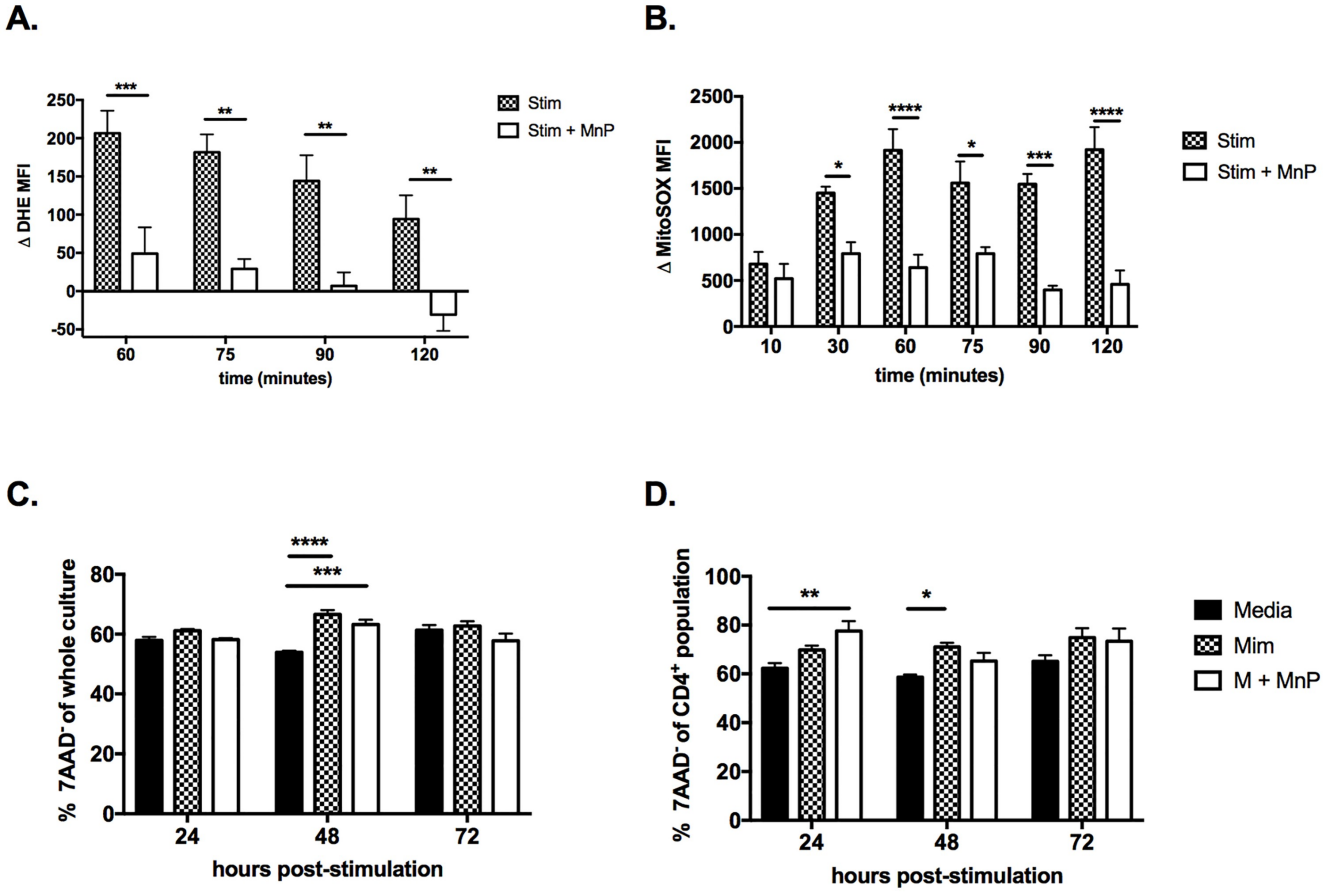
MnP treatment effectively scavenges NADPH oxidase and mitochondrial derived superoxide, while demonstrating no toxicity. NOD splenocytes were pre-treated with or without MnP and then loaded with either Dihydroethidium (DHE; a) or MitoSOX red (b). Splenocytes were stimulated with PMA and ionomycin and read for fluorescence by flow cytometry at the indicated time points. Data are displayed as delta mean fluorescence intensity (Δ MFI) ± SEM calculated as MFI_stimulated_ − MFI_unstimulated_. (c and d) BDC2.5.TCR.Tg splenocyte cultures were stained for 7AAD and CD4 to assess viability of cultures due to MnP treatment. Data are displayed as percent 7AAD^-^ of whole splenocytes (c) and CD4^+^ T cells (d). Significance was determined by Two-way ANOVA with Bonferroni post-hoc analysis of a combined n = 3–5 mice (**** = p<0.0001; *** = p<0.001; ** = p<0.01; *p<0.05).

T cells generate ROS via two sources–a phagocyte-like NADPH oxidase [6, 47] and mitochondrial electron leak [8]. As blockade of each of these sources have differential effects on T cell activation and differentiation, we wanted to further delineate if MnP treatment successfully scavenges ROS from both sources. To do so, the fluorescent indicators dyhidoethidium (DHE) and MitoSOX were utilized as both dyes only fluoresce upon modification by superoxide. DHE measures total superoxide generation, whereas MitoSOX specifically measures that from the mitochondria. Following pre-treatment with either media alone or media with MnP, splenocytes were stimulated with PMA and ionomycin which are known to induce ROS production by T cells [8, 47, 48]. As anticipated, MnP treatment successfully reduced total superoxide generation as measured by DHE (Fig 1A). Additionally, mitochondrial-derived superoxide generation was also diminished by MnP treatment (Fig 1B), indicating that MnP is capable of entering the mitochondria. Together these data reveal that MnP effectively dissipates ROS from both NADPH oxidase and the mitochondria, resulting in reduced total cellular ROS production.

Viability of splenocyte cultures was also assessed to confirm that effects on T cell activation and metabolism were not simply due to MnP toxicity. 7AAD staining results demonstrated no significant difference in viability of whole splenocytes (Fig 1C) nor CD4^+^ T cells (Fig 1D), supporting that the subsequent impact of MnP treatment on T cell metabolic reprogramming is not simply due to agent-associated toxicity.

### Scavenging of ROS during activation halts CD4^+^ T cells at the G_0_/G_1_ checkpoint and inhibits clonal expansion

**Fig 2:**
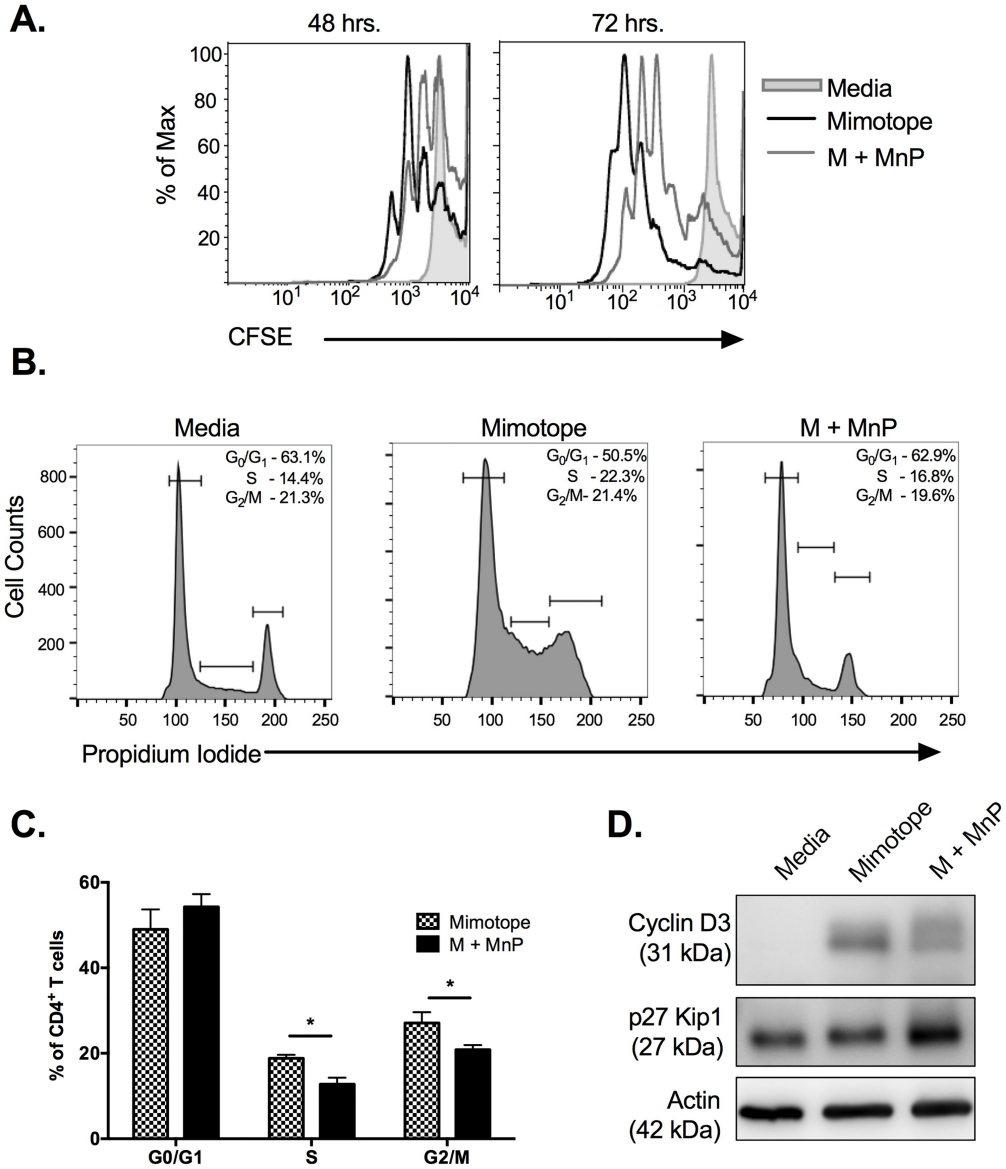
Redox modulation during activation inhibits cell cycle progression of CD4^+^ T cells. NOD.BDC.2.5.TCR.Tg splenocytes were plated in complete splenocyte media and stimulated with 0.05 μM mimotope with or without 68 μM MnP (M + MnP) for 48–72 hours. (a) Prior to stimulation cells were loaded with 1 μM CFSE. Cells were stained with CD4 following harvest and analyzed by flow cytometry for CFSE dilution, indicating proliferation. Unstimulated cells (grey shaded curve) served as negative controls to set proliferation gates. CFSE tracings are representative of n = 6 independent experiments. (b) Cells were fixed, permeabilized, and stained with propidium iodide and CD4-FITC. Cells were analyzed by flow cytometry to determine cell cycle status. CD4^+^ T cells were gated on and cell cycle phases were set based upon unstimulated controls (left panel). (c) Percentages of n = 5 experiments were combined and graphed as mean ± SEM (* = p<0.05). (d) 48 hrs. post-stimulation, cells were harvested and analyzed by Western blot for p27 Kip1 and Cyclin D3 expression. β actin expression served as the loading control.

Acute doses of ROS are required for cell cycle progression and proliferation of CD4^+^ T cells [49, 50]. Therefore, we wanted to examine if scavenging of ROS via MnP treatment inhibited this process during activation. Here, we utilized the NOD.BDC.2.5.TCR.Tg (BDC.2.5) mouse model, in which the CD4^+^ T cells of this animal have been shown to demonstrate diabetogenic potential [51]. CD4^+^ T cells from BDC.2.5 animals stimulated with their specific antigen mimotope (M), demonstrated high proliferative capacity as demonstrated by CFSE dilution (Fig 2A). ROS inhibition via MnP treatment during antigen-dependent stimulation resulted in CD4^+^ T cells undergoing fewer rounds of proliferation at both 48 and 72 hrs post-stimulation as compared to stimulation alone (Fig 2A). CD4^+^ T cells treated with MnP did show low levels of proliferation, which was not surprising as MnP does not fully block all ROS production (Fig 1A and 1B).

Since overall proliferation was reduced due to MnP treatment, we wanted to examine if CD4^+^ T cells were being arrested at a specific cell cycle checkpoint. Consequently, propidium iodide staining was used to examine the distribution of CD4^+^ T cells in the various phases of the cell cycle–G_0_/G_1_, S, and G_2_/M. By 48 hours post-stimulation, significantly fewer CD4^+^ T cells had progressed to the later stages of the cell cycle (S phase and G_2_/M phase) following MnP treatment, as compared to stimulated controls (Fig 2B and 2C; p<0.05). These results correlated with increased expression of the cell cycle inhibitor p27 Kip1 (Fig 2D), which is responsible for impeding progression from G_1_ to S phase [52]. Additionally, protein analysis demonstrated reduced expression of Cyclin D3, a promoter of S phase transition, due to MnP treatment, again indicating limited cell cycle progression (Fig 2D). p27 Kip1 is also a potent Cyclin D3 inhibitor [53], and therefore, its maintained expression results in cell cycle arrest. Together, these results indicate that dissipating ROS during activation halts CD4^+^ T cells at the G_1_/S checkpoint in the cell cycle via maintaining p27 Kip1 expression.

### Transition to aerobic glycolysis during CD4^+^ T cell activation is dependent upon cellular redox status

CD4^+^ T cells undergo massive metabolic reprogramming during the transition from naïve to effector states [15, 54], and in order to support this metabolic reprogramming, CD4^+^ T cells must upregulate various glycolytic enzymes, transcriptional regulators, and glucose transporters [15]. Impeding this process results in reduced clonal expansion [17], effector function [55], and overall T cell responses in disease models [11]. Proliferation and metabolism are two tightly coupled cellular processes, and inhibition of glycolysis has been shown to result in diminished proliferation [10]. Therefore, we hypothesized that as ROS scavenging diminished CD4^+^ T cell proliferation (Fig 2), it may be doing so by modulating metabolic reprogramming.

**Fig 3:**
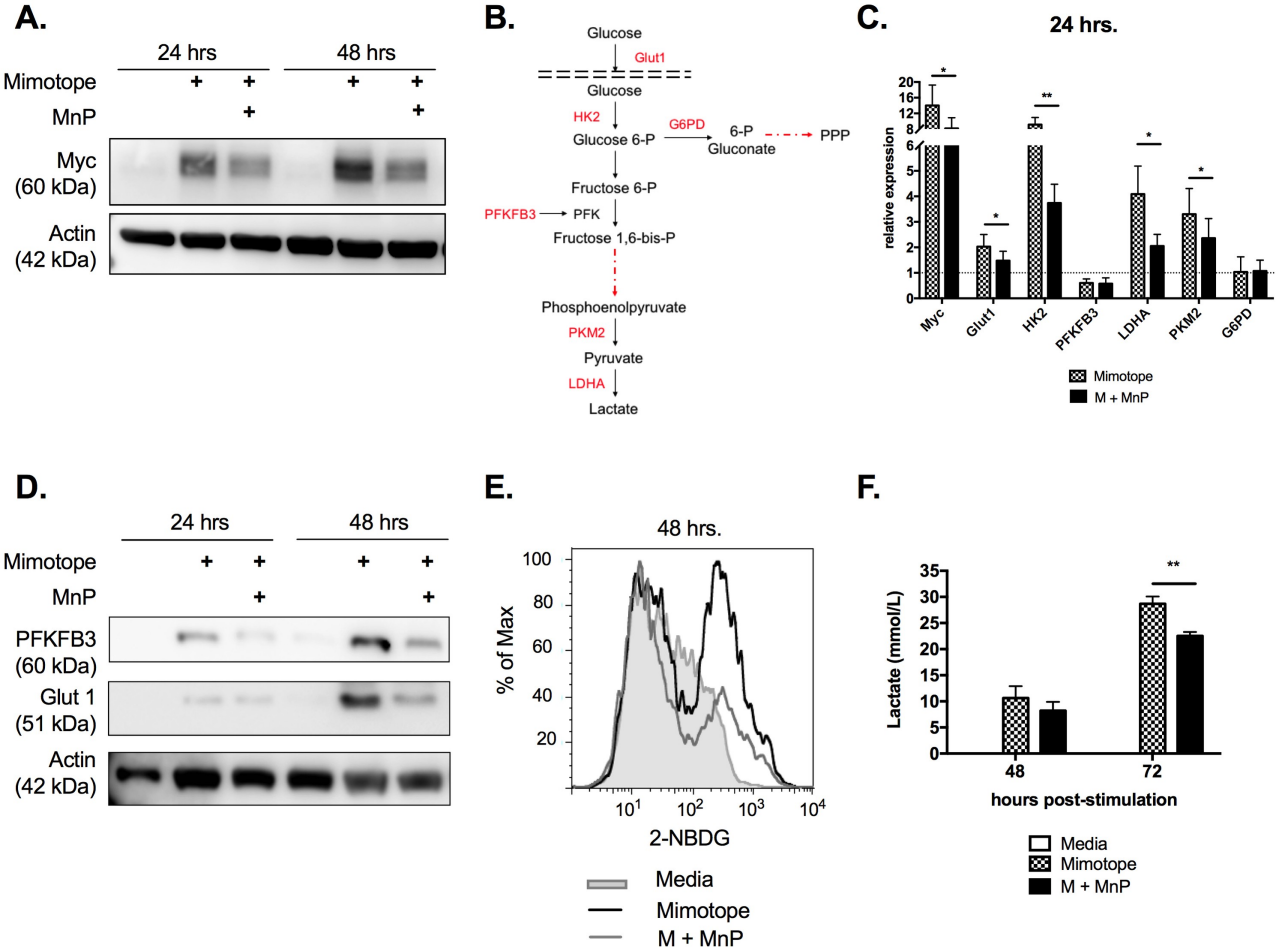
Transition to aerobic glycolysis during CD4^+^ T cell activation is dependent upon cellular redox status. (a) Protein lysates were probed for Myc expression. β actin expression served as the loading control. (b) Glycolytic pathway with assayed Myc-dependent genes indicated in red. (c) 24 hours post-stimulation, mRNA expression of various glycolysis-associated genes was assessed by qRT-PCR. Relative values were normalized to unstimulated, naïve controls (Media). Data displayed are means of n = 5 independent experiments ± SEM. (d) Western blot analysis of specific Myc targets, PFKFB3 and Glut1. (e) 48 hours post-stimulation, cells were treated with 100 μM 2-NBDG and stained for CD4 following incubation. Glucose uptake was measured as 2-NBDG fluorescence of the CD4^+^ population. Histograms are representative of n = 8 experiments. (f) Lactate was measured in culture supernatants using the Accutrend Plus meter (Roche). Data are graphed as means of n = 5 experiments ± SEM. Statistical significance between mimotope and M + MnP groups was calculated using a Student’s t test (* = p<0.05, ** = p<0.01).

The transcription factor Myc has been shown to be critical for promoting aerobic glycolysis in both CD4^+^ T cells and tumors alike, by initiating expression of various glycolytic enzymes and glucose transporters [15, 16], along with driving cell cycle progression [16]. Protein and quantitative RT-PCR analysis of *in vitro* stimulated BDC2.5.TCR.Tg splenocytes confirmed increased Myc expression at 24 and 48 hours post-stimulation (Fig 3A and 3C). In contrast, MnP treatment resulted in contracted Myc upregulation at both time points (Fig 3A and 3C). These results correlated with reduced mRNA expression of several Myc-dependent genes necessary for aerobic glycolysis including glut1, hexokinase 2 (HK2), lactate dehydrogenase A (LDHA), and pyruvate kinase M2 (PKM2) (Fig 3B and 3C; p<0.05).

Following gene expression analysis, we sought to investigate protein expression of two key Myc-dependent targets–Glut1, the glucose transporter expressed by activated T cells that has the greatest impact on differentiation [56–58]; and, PFKFB3, a rate limiting enzyme that aids in committing glucose to being metabolized via glycolysis [59]. Also, modulation of activity and expression of PFKFB3 in autoreactive T cells has been shown to ameliorate disease, suggesting PFKFB3 as a potential therapeutic target for autoimmunity [59, 60]. As anticipated, protein expression of Glut1 was reduced in MnP-treated splenocytes, further corroborating qRT-PCR results (Fig 3C). However, while qRT-PCR results did not show differences in mRNA expression of the enzyme PFKFB3 (Fig 3D), protein analysis did show reduced upregulation due to MnP scavenging ROS (Fig 3D). These results underscore the importance ROS play in enabling the transcriptional alterations necessary for aerobic glycolysis.

As glucose is the primary substrate for aerobic glycolysis, and our data indicated that MnP treatment inhibited Glut1 expression, we wanted to measure the effect of MnP treatment on glucose uptake. To do so, splenocyte cultures were incubated with the fluorescent glucose analog 2-NBDG, and uptake was measured by flow cytometry. As with diminished Glut1 expression, MnP treatment resulted in reduced glucose uptake by CD4^+^ T cells (Fig 3E). These results correlated with reduced lactate production, the byproduct of aerobic glycolysis, as measured in culture supernatants, further indicating a significant reduction in the utilization of aerobic glycolysis due to redox modulation (Fig 3F; p<0.01). Overall, these data ascertain that alterations in CD4^+^ T cell redox status limits upregulation and utilization of the glycolytic pathway necessary for driving effector differentiation and clonal expansion.

### mTOR signaling is reduced upon treatment with MnP

The mammalian target of rapamycin (mTOR) pathway has been shown to be pivotal in driving metabolic changes during T cell activation and regulating CD4^+^ T cell lineage commitment [61, 62]. Studies examining the effects of rapamycin on T cell metabolism have indicated that inhibition of mTOR activation and signaling during T cell activation resulted in diminished proliferation, effector function, and glycolytic capacity [19]. Given the fact that MnP treatment inhibited cell cycle progression (Fig 2) and aerobic glycolysis (Fig 3), and that the mTOR pathway is critical in mediating both of these processes, we sought to measure mTOR activation and its downstream signaling.

**Fig 4:**
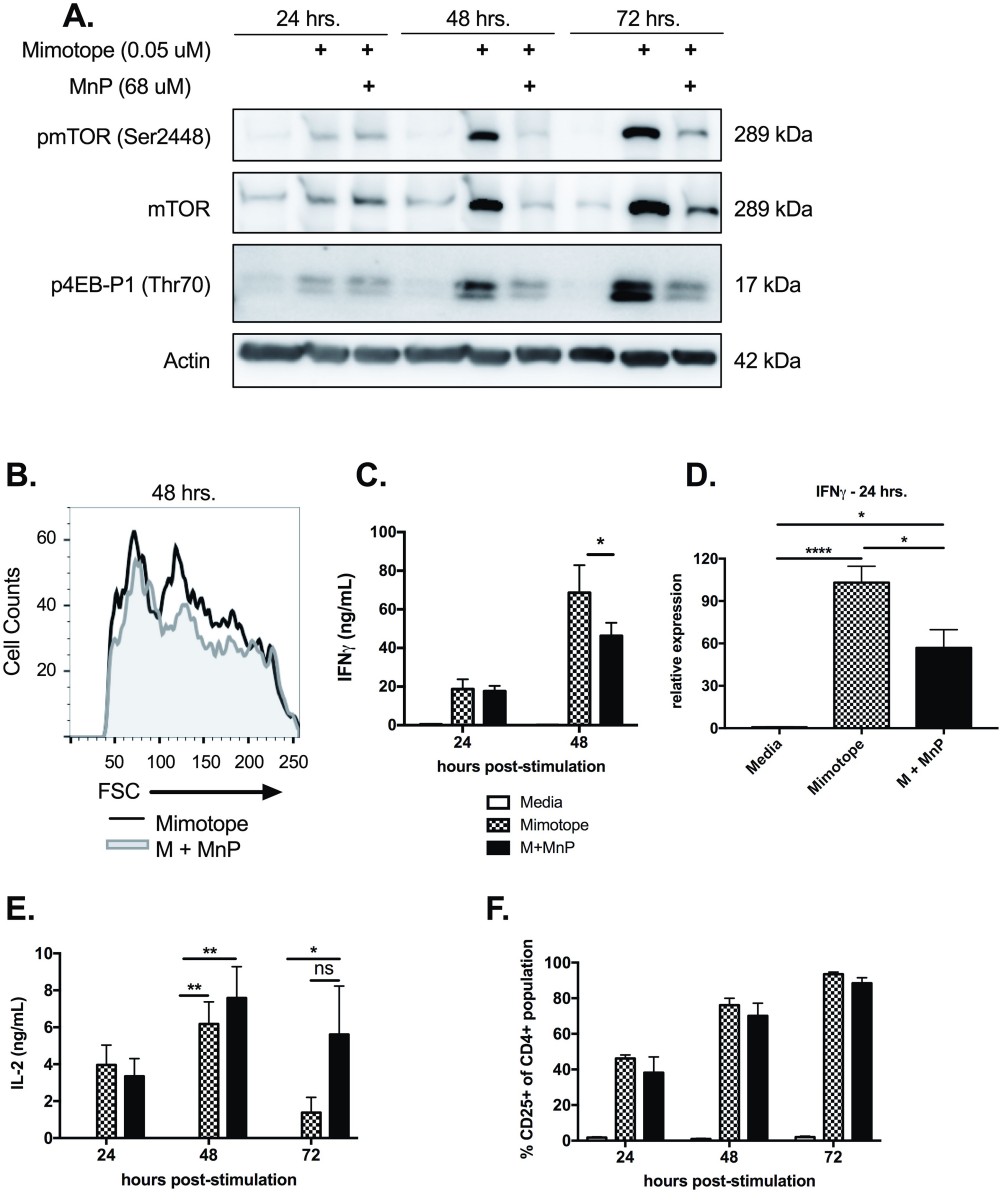
mTOR signaling is inhibited upon MnP treatment. (a) Western blot analysis for phosphorylated mTOR (Ser4998; active), total mTOR, and the downstream mTOR target, phosphorylated 4E-BP1 (Thr70). β actin expression served as the loading control. (b) Forward scatter (FSC) of CD4^+^ T cells was measured by flow cytometry as an indication of cell blasting. Histogram is representative of n = 5 experiments. (c) IFNγ secretion was assessed by ELISA of culture supernatants. (d) Analysis of *Ifnγ* gene expression by qRT-PCR at 24 hours post-stimulation. (e) ELISA results measuring IL-2 secretion from culture supernatants. (e) Frequency of CD4^+^ T cells expressing CD25 following activation. Data displayed are combined means ± SEM of n = 6–9 experiments. Statistical significance was calculated using either a one-way or two-way ANOVA with Tukey or Bonferroni post-hoc analysis, respectively. (* = p<0.05). Media alone treated splenocytes served as negative controls.

Protein analysis of *in vitro* stimulations indicated that MnP treatment during mimotope stimulation resulted in reduced total mTOR expression and also activation, as measured by phosphorylation at Ser2448 at 48 and 72 hours post-stimulation (Fig 4A). We also examined phosphorylation of mTOR’s downstream target 4E-BP1, a translational repressor that is inhibited upon hyperphosphorylation [18]. Reduced phosphorylation of 4E-BP1 (Thr70) was exhibited in MnP treated CD4^+^ T cells, as compared to stimulated controls (Fig 4A), indicating reduced mTOR signaling.

In addition to driving activation, mTOR signaling is also necessary for mediating the growth phase of mammalian cells in preparation for expansion [63] and driving effector differentiation in T cells [18]. As anticipated, with reduced mTOR signaling, MnP-treated CD4^+^ T cells demonstrated reduced growth at 48 hours as measured by forward scatter (Fig 4B), and reduced IFNγ secretion (Fig 4C; p<0.05). Additionally, we assessed IFNγ mRNA expression by splenocytes 24 hours following stimulation. qRT-PCR results indicated that MnP treatment during stimulation hindered upregulation of IFNγ mRNA as compared to mimotope stimulation alone (Fig 4D; p<0.05), suggesting that the reduced secreted levels due to ROS scavenging was not simply due to reduced proliferation and the presence of fewer T cells. Overall, these results indicate that ROS signaling is required for amplifying mTOR signaling upon activation, which then enables optimal T cell proliferation (Figs 2 and 4) and glycolysis (Figs 3 and 4).

We also examined Interleukin-2 (IL-2) levels in our *in vitro* cultures, as IL-2 is essential for aiding T cell clonal expansion. Interestingly, we saw no defect in IL-2 production due to MnP treatment as compared to stimulation alone at all time points examined (Fig 4E). Additionally, there was no difference in expression of the high-affinity IL-2 receptor subunit, CD25 (Fig 4F). These results suggest that while redox modulation has no effect on the production of IL-2 or receptor expression, it does alter downstream signaling as mTOR signaling is, in part, IL-2 driven.

### Inhibition of T cell generated ROS reduces proliferation, growth and glucose uptake

Third signal ROS are necessary for driving T cell activation, and our data here indicate they are also critical for facilitating metabolic reprogramming (Figs 3 and 4). Previous work has revealed that both APCs and T cells express functional NOX enzymes that are activated during MHC-TCR engagement, resulting in ROS production from both the APC and T cell [47, 48, 64]. Therefore, we sought to further delineate if T cell-derived ROS were alone sufficient for mediating the transition to aerobic glycolysis during activation, and if MnP treatment would inhibit this process.

**Fig 5:**
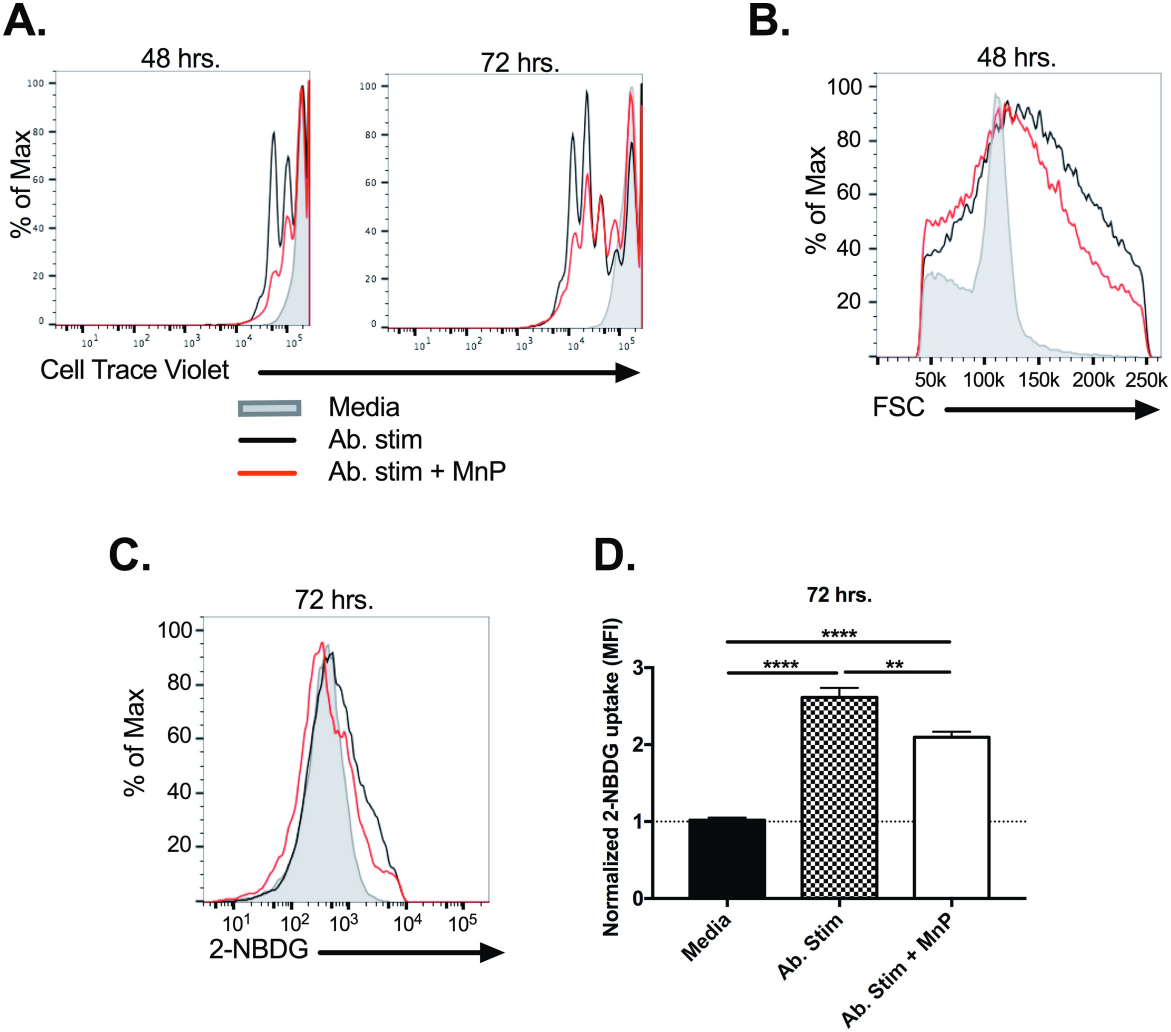
Inhibition of CD4^+^ T cell-derived ROS during activation results in reduced proliferation, growth, and glucose uptake. CD4^+^ T cells were isolated from whole spleens using magnetic bead isolation kits and stimulated with plate-bound αCD3 and αCD28. (a) Representative proliferation tracings of CD4^+^ T cells at 48 and 72 hrs post-stimulation. (b) Forward scatter (FSC) of CD4^+^ T cells as measured by flow cytometry. (c) Representative glucose uptake histograms (2-NBDG MFI) at 72 hrs post-stimulation. (d) Fold increase of glucose uptake as compared to T cells treated with media alone. Data were normalized to controls within experiments and are presented as mean ± SEM of n = 6 combined experiments. Statistical significance was calculated using a One-way ANOVA with Tukey post-hoc analysis. (** = p<0.01; **** = p<0.0001). Histograms are representative of n = 6 independent experiments.

To do so, CD4^+^ T cells were isolated from whole splenocytes and stimulated *in vitro* with plate-bound αCD3/αCD28, with or without MnP. Flow cytometric analysis of stimulated T cells indicated that ROS inhibition by MnP resulted in reduced proliferation at both 48 and 72 hours post-stimulation as compared to activated T cells not treated with MnP (Fig 5A). ROS scavenging also resulted in reduced cell growth during activation as measured by forward scatter (Fig 5B) and glucose uptake at 72 hours post-stimulation (Fig 5C and 5D; p<0.01). These results are in accordance with data presented in Figs 2 and 4, and further elucidate that the role of ROS in T cell transition to aerobic glycolysis is not specific to APC-derived ROS.

### MnP treatment enhances activation of AMPK, a known mTOR inhibitor

AMPK, or adenosine monophosphate activated protein kinase, is a nutrient sensor shown to be responsible for driving oxidative metabolism due to ATP depletion, and it becomes active following phosphorylation of its catalytic α subunit [21, 65, 66]. The antioxidant resveratrol has been shown to activate AMPK [20], and AMPK is an established negative regulator of the Warburg effect (aerobic glycolysis) [22] and mTOR signaling [23, 65]. Since our data indicated that MnP treatment inhibits mTOR-driven aerobic glycolysis in CD4^+^ T cells, and MnP is a potent antioxidant [32, 33], we hypothesized that MnP treatment would result in AMPK activation, thereby inhibiting mTOR signaling and aerobic glycolysis.

**Fig 6:**
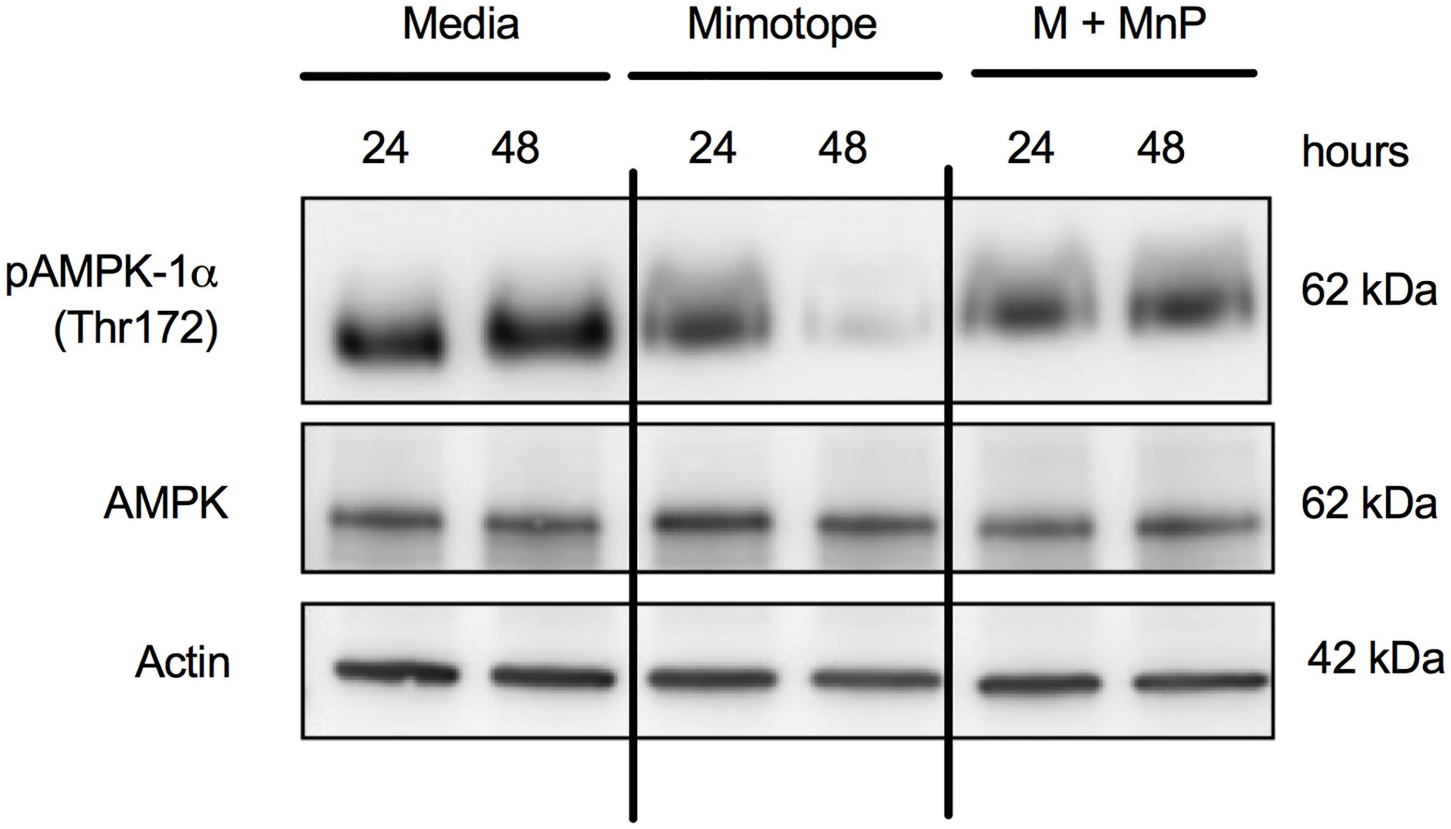
Alteration in redox status of CD4^+^ T cells maintains AMPK activation. (a) Protein lysates were probed with antibodies for phosphorylated AMPK-1α (Thr172; activated), total AMPK, and β actin (loading control). Data are a representative of n = 5 independent experiments.

**Fig 7:**
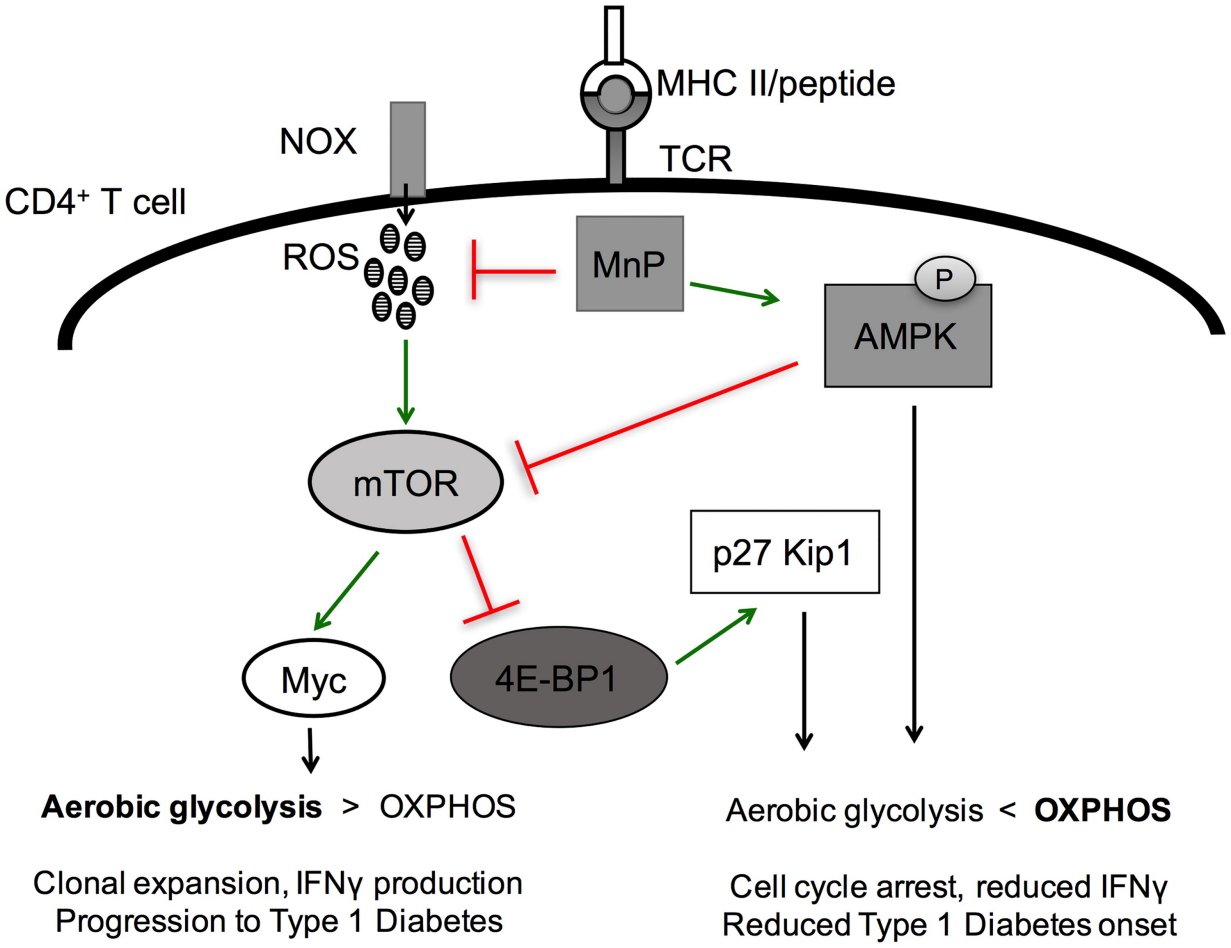
Mechanism for the effect of ROS inhibition on CD4^+^ T cells during activation-induced metabolic reprogramming. Upon T cell receptor–MHC interaction, ROS are generated by a functional NOX expressed by T cells. These ROS serve as signaling molecules to help propagate mTOR signaling resulting in Myc upregulation and progression to aerobic glycolysis. Treatment with the ROS-scavenging and potent antioxidant results in inhibition of ROS and maintains potent AMPK activation; thereby, inhibiting mTOR via a two-pronged approach, stabilizing OXPHOS, and limiting T cell proliferation.

AMPK is highly activated in naïve T cells, and protein analysis demonstrated that the levels of AMPK phosphorylation (Thr172) due to MnP treatment were comparable to those of media-treated, naïve cells, as indicated by a relative expression value of 1 (Fig 6). Mimotope stimulation alone resulted in reduced AMPK phosphorylation at 48 hours as compared to both media and MnP-treated splenocytes (Fig 6). These results reveal that MnP treatment may not enhance AMPK activation, but rather maintain active levels even in the presence of antigenic stimulation, thus impeding mTOR signaling and aerobic glycolysis (Fig 7).

### Inhibition of aerobic glycolysis due to redox modulation limits the diabetogenic potential of autoreactive CD4^+^ T cells

**Fig 8:**
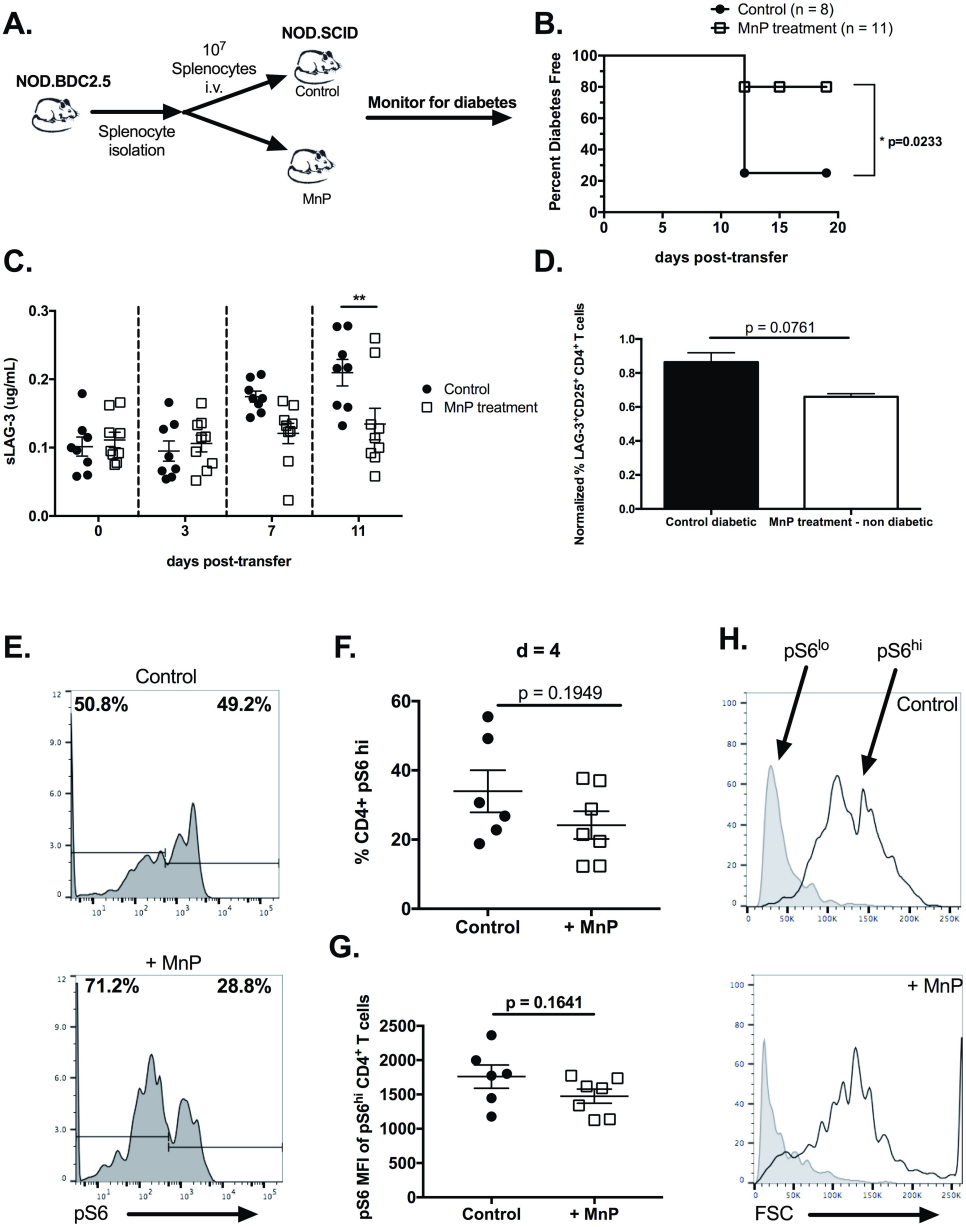
Reducing glycolytic capacity by redox modulation inhibits diabetogenic potential of CD4^+^ T cells. (a) Schematic of adoptive transfer model of T1D. (b) Survival curve of diabetes free animals following adoptive transfer. Animals were deemed diabetic following 2 consecutive blood glucose readings of >350 mg/dL. Statistical significance of disease progression was calculated using a Kaplan-Meier test (* = p<0.05). (c) T cell activation and prediction of diabetes was measured by serum levels of sLAG-3 by ELISA. Statistical significance was calculated using a Two-way ANOVA with Bonferroni post-hoc analysis. (** = p<0.01). (d) Normalized percent of LAG-3^+^CD25^+^CD4^+^ T cells from spleens of control diabetic and MnP-treated non-diabetic animals (n = 6–8 animals per group; p = 0.0761). (e) Mean fluorescence intensity of pS6 in peripheral blood CD4^+^ T cells from animals at day 4 post-transfer. (f) Representative histograms of S6 phosphorylation from peripheral blood CD4^+^ T cells at day 4 post-transfer from control and MnP treated animals. (g-h) Quantification of frequency of pS6^hi^ and pS6^lo^ CD4^+^ T cells. (i) Forward scatter analysis of pS6^lo^ and pS6^hi^ CD4^+^ T cells.

In order to test if inhibiting progression to aerobic glycolysis in CD4^+^ T cells delayed diabetes incidence, an adoptive transfer model was used (Fig 8A). Here, BDC.2.5.TCR.Tg splenocytes were isolated and transferred i.v. into non-diabetic NOD.*scid* recipients. A cohort of the recipient animals was treated with 10 mg/kg MnP starting the day prior to adoptive transfer as described in the methods. 80% of untreated controls exhibited fulminant diabetes by 12 days post-transfer (Fig 8B); however, MnP treatment inhibited diabetes progression, with only 20% of treated animals becoming diabetic throughout the duration of the experiment (Fig 8B; p = 0.0233).

We and others have reported that increased serum levels of the inhibitory receptor Lymphocyte Activation Gene-3 (LAG-3) due to its redox-dependent cleavage from the surface of T cells, serves as a viable marker of T cell activation [67, 68] and conceivable predictor of T1D [35]. Therefore, we measured soluble LAG-3 (sLAG-3) in the serum of recipient animals at various time points post-transfer, as a means of measuring T cell activation. At day 7 post-transfer, while all animals were euglycemic, control animals presented with elevated serum levels of sLAG-3 as compared to those treated with MnP (Fig 8C). Those controls went on to become diabetic by day 11 post-transfer, while animals treated with MnP did not (p<0.01). Together, these data demonstrate that sLAG-3 serum levels, as a measure of T cell activation, predicts diabetes onset, and MnP treatment controls this redox-dependent process.

Splenocytes from recipient animals were analyzed by flow cytometry to assess T cell activation *in vivo*. Two markers of activation were measured: CD25, the IL-2 high affinity receptor, and LAG-3 (Fig 8D), as both markers are more highly expressed on the surface of activated CD4^+^ T cells than on naïve T cells [69, 70]. CD4^+^ T cells from diabetic controls displayed increased frequency of CD25^+^LAG-3^+^ double positive T cells, indicative of an activated phenotype, in comparison to CD4^+^ T cells from MnP treated non-diabetic animals (Fig 8D; p = 0.0761).

As *in vitro* results indicated reduced mTOR signaling (Fig 5), we also wanted to confirm these results *in vivo*. Peripheral blood samples were taken at day 4 post-adoptive transfer and levels of phosphorylated S6 ribosomal protein (S6), an mTOR target, were measured by flow cytometry. Results indicated two populations of CD4^+^ T cells with respect to pS6 expression, CD4^+^pS6^hi^ T cells and CD4^+^pS6^lo^ T cells (Fig 7E). CD4^+^ T cells from MnP treated animals exhibited a lower frequency of pS6^hi^ cells (Fig 8E and 8F), as compared to T cells from control animals, indicating reduced T cell activation due to MnP treatment. Moreover, pS6 expression (as measured by mean fluorescence intensity) was increased in CD4^+^pS6^hi^ T cells from control animals compared to the MnP-treated cohort (Fig 8G). These results suggest that those T cells that do receive some activation signal despite the presence of MnP, are still unable to induce mTOR signaling to the extent of stimulated controls. Lastly, studies from *Pollizzi et al*. demonstrated that T cells with high levels of mTOR signaling during activation, were larger than those with lower mTOR activation [71]. In accordance with these and other studies, CD4^+^pS6^hi^ T cells were larger than the CD4^+^pS6^lo^ T cells, regardless of treatment (Fig 8H). Yet, as with higher mTOR signaling induction in control T cells (Fig 8G), pS6^hi^ T cells from these animals were also larger than those treated with MnP (Fig 8H), supporting their finding that T cell size is directly related to mTOR activity and activation [72]. These results demonstrate that MnP treatment *in vivo* alters mTOR signaling during T cell activation, resulting in reduced CD4^+^ T cell diabetogenicity.

## Discussion

Work from our laboratory and others has demonstrated that acute doses of ROS are required for mediating T cell activation, and that ROS inhibition results in dampened T cell responses [6–8, 35, 37]. Treatment with a manganese metalloporphyrn (MnP) successfully scavenges a significant amount of the ROS produced, with no toxicity to the T cells themselves (Fig 1). Cell cycle entry and proliferation have been shown to be highly dependent upon ROS signaling, as many cyclin-dependent kinases that mediate cell cycle progression, and cell cycle inhibitors are known to be redox sensitive [49]. Indeed, our results indicated that dissipating ROS by MnP resulted in reduced cell cycle entry via maintenance of the cell cycle inhibitor p27 Kip1 (Fig 2). Interestingly, T cell proliferation and effector function were not completed ablated upon MnP treatment, potentially since MnP treatment did not completely dissipate all superoxide generated upon T cell activation (Fig 1A and 1B). Additionally, completely depleting the cell of all ROS would result in toxicity. These results highlight that fine-tuning of ROS signaling has dramatic effects on T cell outcome.

In order to transition to an effector after activation, naïve CD4^+^ T cells must undergo massive reprogramming at the metabolic level, transitioning from oxidative phosphorylation to aerobic glycolysis [10–12]. Aerobic glycolysis, or the Warburg effect, is required for supporting optimal T cell clonal expansion and macromolecule synthesis, yet how ROS affect metabolic reprogramming during T cell activation remains poorly understood. Here, our data indicate that ROS are necessary for driving optimal mTOR signaling (Fig 5A) and upregulation of the transcription factor Myc (Fig 3A), two key players that have roles in coordinating both aerobic glycolysis and cell cycle entry [16, 18, 73]. It has been reported that T cell effector function is tightly regulated by both aerobic glycolysis and cell cycle. In fact, proliferation and IFNγ production have a direct relationship–as rounds of proliferation increase so does IFNγ production [74] Our results reiterate these findings in that redox modulation inhibits aerobic glycolysis (Fig 3) and proliferation (Fig 2), concomitant with IFNγ secretion (Fig 5C). Also, these findings suggest that redox reactions in fact supersede these pathways.

A well described characteristic of T cells is that they divide asymmetrically, generating two distinctly different daughter cells. These daughter cells exhibit differential mTOR activation, cellular metabolism, and eventual differentiation [71, 75, 76]. Specifically, the larger, mTOR^hi^, glycolytic daughters demonstrate a more T effector phenotype, whereas the smaller, mTOR^lo^, oxidative daughters are more memory-like [71, 72, 75]. Interestingly, redox modulation skewed T cells towards a higher percentage of mTOR^lo^ T cells as compared to untreated controls (Fig 8E–8H), which may suggest a role for redox in asymmetric division. It is also plausible that scavenging of ROS is simply inhibiting activation and the pS6^lo^ T cells are those that remain naïve. Also in this report, *Pollizzi et al*. indicated that there was no difference in CD25 surface expression between mTOR^hi^ and mTOR^lo^ T cells [72]. Even with CD25 expression and the presence of IL-2, mTOR^lo^ T cells failed to proliferate as robustly [63], which is in accordance with the *in vitro* results demonstrated (Fig 5D and 5E), suggesting some additional regulatory mechanism(s) at play. It seems plausible that ROS could be a contributing factor in mediating T cell asymmetry; however, further studies would be necessary.

As mentioned, one of the downstream targets of mTOR is 4E-BP1, a translational repressor [63]. For translation to ensue, hyperphosphorylation of 4E-BP1 by mTOR is critical for inhibition of the repressor. Since our data indicate a decrease in phosphorylation or inhibition of 4E-BP1, it is likely that maintenance of the repressor contributed to the reduced protein expression of Glut1 and PFKFB3 (Fig 3E). This, coupled with reduced Myc expression, may synergistically impede metabolic reprogramming. With regards to cell cycle progression, inhibition of 4E-BP1 by mTOR is required for promoting expression of Cyclin D3 [19]. Additionally, mTOR signaling has been shown to lead to increased p27 Kip1 degradation as a means of supporting cell cycle progression [19]. Therefore, MnP-mediated decreased 4E-BP1 hyperphosphorylation likely contributed to decreased Cyclin D3 expression and decreased p27 Kip1 degradation (Fig 2D), promoting cell cycle arrest.

CD4^+^ T cells are a primary mediator of immunopathology in T1D; therefore, we wanted to determine if inhibiting metabolic reprogramming by MnP treatment reduced their diabetogenic potential. As anticipated, modulating the CD4^+^ T cell glycolytic rate via MnP delayed T1D onset in an adoptive transfer model (Fig 8B). T1D is known to be highly driven by free radicals. Not only does oxidative stress result in islet beta cell death, but it also serves to activate and mobilize inflammatory macrophages and T cells, driving even more immunopathology. Therefore, further outlining the mechanisms in which these molecules influence immune cells is vital. More recent studies in other autoimmune diseases like systemic lupus and rheumatoid arthritis have helped to delineate potential for metabolic-based therapies in ameliorating disease [25, 26, 60], further supporting the need to more fully understand mechanisms governing immune cell metabolism. It is worth noting that in rheumatoid arthritis, pathogenic T cells were shown to produce increased levels of ROS that resulted in glycolysis inhibition and increased apoptosis [77]. Consequently, ROS may play different roles depending on the disease context, making it even more imperative to further understand their influence.

In summary, we have demonstrated that ROS inhibition by a manganese metalloporphyrin during diabetogenic CD4^+^ T cell activation is capable of impeding the metabolic transition from oxidative phosphorylation to aerobic glycolysis necessary for optimal T cell responses. We propose a model in which ROS and cellular redox balance is critical for amplification of the mTOR/Myc pathway, and thus aerobic glycolysis (Fig 7). These findings present potential implications in tempering T cell responses in autoimmunity, and also controlling tumor metabolism and cell growth in cancer.

## References

[1] Achenbach P, Bonifacio E, Koczwara K, Ziegler AG. Natural history of type 1 diabetes. Diabetes. 2005;54 Suppl 2:S25–31. 16306336 10.2337/diabetes.54.suppl_2.s25

[2] van Belle TL, Coppieters KT, von Herrath MG. Type 1 diabetes: etiology, immunology, and therapeutic strategies. Physiological reviews. 2011;91(1):79–118. 10.1152/physrev.00003.2010 21248163

[3] Delmastro MM, Piganelli JD. Oxidative stress and redox modulation potential in type 1 diabetes. Clin Dev Immunol. 2011;2011:593863 10.1155/2011/593863 21647409 PMC3102468

[4] Haskins K, Bradley B, Powers K, Fadok V, Flores S, Ling X, et al Oxidative stress in type 1 diabetes. Annals of the New York Academy of Sciences. 2003;1005:43–54. 14679039 10.1196/annals.1288.006

[5] Panday A, Sahoo MK, Osorio D, Batra S. NADPH oxidases: an overview from structure to innate immunity-associated pathologies. Cell Mol Immunol. 2015;12(1):5–23. 10.1038/cmi.2014.89 25263488 PMC4654378

[6] Jackson SH, Devadas S, Kwon J, Pinto LA, Williams MS. T cells express a phagocyte-type NADPH oxidase that is activated after T cell receptor stimulation. Nature immunology. 2004;5(8):818–27. Epub 2004/07/20. 10.1038/ni1096 15258578

[7] Tse HM, Thayer TC, Steele C, Cuda CM, Morel L, Piganelli JD, et al NADPH oxidase deficiency regulates Th lineage commitment and modulates autoimmunity. Journal of immunology. 2010;185(9):5247–58. Epub 2010/10/01. 10.4049/jimmunol.1001472 PMC3190397 20881184

[8] Sena LA, Li S, Jairaman A, Prakriya M, Ezponda T, Hildeman DA, et al Mitochondria are required for antigen-specific T cell activation through reactive oxygen species signaling. Immunity. 2013;38(2):225–36. Epub 2013/02/19. 10.1016/j.immuni.2012.10.020 23415911 PMC3582741

[9] Curtsinger JM, Schmidt CS, Mondino A, Lins DC, Kedl RM, Jenkins MK, et al Inflammatory cytokines provide a third signal for activation of naive CD4+ and CD8+ T cells. Journal of immunology. 1999;162(6):3256–62. 10092777

[10] Pearce EL, Poffenberger MC, Chang CH, Jones RG. Fueling immunity: insights into metabolism and lymphocyte function. Science. 2013;342(6155):1242454 10.1126/science.1242454 24115444 PMC4486656

[11] MacIver NJ, Michalek RD, Rathmell JC. Metabolic regulation of T lymphocytes. Annual review of immunology. 2013;31:259–83. Epub 2013/01/10. 10.1146/annurev-immunol-032712-095956 23298210 PMC3606674

[12] Michalek RD, Rathmell JC. The metabolic life and times of a T-cell. Immunological reviews. 2010;236:190–202. Epub 2010/07/20. 10.1111/j.1600-065X.2010.00911.x 20636818 PMC2983473

[13] Palmer CS, Cherry CL, Sada-Ovalle I, Singh A, Crowe SM. Glucose Metabolism in T Cells and Monocytes: New Perspectives in HIV Pathogenesis. EBioMedicine. 2016;6:31–41. 10.1016/j.ebiom.2016.02.012 27211546 PMC4856752

[14] Kim JW, Dang CV. Cancer's molecular sweet tooth and the Warburg effect. Cancer research. 2006;66(18):8927–30. Epub 2006/09/20. 10.1158/0008-5472.CAN-06-1501 16982728

[15] Wang R, Dillon CP, Shi LZ, Milasta S, Carter R, Finkelstein D, et al The transcription factor Myc controls metabolic reprogramming upon T lymphocyte activation. Immunity. 2011;35(6):871–82. Epub 2011/12/27. 10.1016/j.immuni.2011.09.021 22195744 PMC3248798

[16] Dang CV. MYC, metabolism, cell growth, and tumorigenesis. Cold Spring Harbor perspectives in medicine. 2013;3(8). 10.1101/cshperspect.a014217 PMC3721271 23906881

[17] Macintyre AN, Rathmell JC. Activated lymphocytes as a metabolic model for carcinogenesis. Cancer & Metabolism. 2013;1(5). 10.1186/2049-3002-1-5 PMC3834493 24280044

[18] Waickman AT, Powell JD. mTOR, metabolism, and the regulation of T-cell differentiation and function. Immunological reviews. 2012;249(1):43–58. Epub 2012/08/15. 10.1111/j.1600-065X.2012.01152.x 22889214 PMC3419491

[19] Powell JD, Pollizzi KN, Heikamp EB, Horton MR. Regulation of immune responses by mTOR. Annual review of immunology. 2012;30:39–68. 10.1146/annurev-immunol-020711-075024 22136167 PMC3616892

[20] Price NL, Gomes AP, Ling AJ, Duarte FV, Martin-Montalvo A, North BJ, et al SIRT1 is required for AMPK activation and the beneficial effects of resveratrol on mitochondrial function. Cell metabolism. 2012;15(5):675–90. 10.1016/j.cmet.2012.04.003 22560220 PMC3545644

[21] Woods A, Johnstone SR, Dickerson K, Leiper FC, Fryer LG, Neumann D, et al LKB1 is the upstream kinase in the AMP-activated protein kinase cascade. Curr Biol. 2003;13(22):2004–8. 14614828 10.1016/j.cub.2003.10.031

[22] Faubert B, Boily G, Izreig S, Griss T, Samborska B, Dong Z, et al AMPK is a negative regulator of the Warburg effect and suppresses tumor growth in vivo. Cell metabolism. 2013;17(1):113–24. Epub 2013/01/01. 10.1016/j.cmet.2012.12.001 23274086 PMC3545102

[23] Zheng Y, Delgoffe GM, Meyer CF, Chan W, Powell JD. Anergic T cells are metabolically anergic. Journal of immunology. 2009;183(10):6095–101. 10.4049/jimmunol.0803510 PMC2884282 19841171

[24] Jang M, Kim SS, Lee J. Cancer cell metabolism: implications for therapeutic targets. Exp Mol Med. 2013;45:e45 10.1038/emm.2013.85 24091747 PMC3809361

[25] Yin Y, Choi SC, Xu Z, Perry DJ, Seay H, Croker BP, et al Normalization of CD4+ T cell metabolism reverses lupus. Sci Transl Med. 2015;7(274):274ra18 10.1126/scitranslmed.aaa0835 25673763 PMC5292723

[26] Yin Y, Choi SC, Xu Z, Zeumer L, Kanda N, Croker BP, et al Glucose Oxidation Is Critical for CD4+ T Cell Activation in a Mouse Model of Systemic Lupus Erythematosus. J Immunol. 2016;196(1):80–90. 10.4049/jimmunol.1501537 26608911 PMC4684991

[27] Mauro C, Leow SC, Anso E, Rocha S, Thotakura AK, Tornatore L, et al NF-kappaB controls energy homeostasis and metabolic adaptation by upregulating mitochondrial respiration. Nat Cell Biol. 2011;13(10):1272–9. 10.1038/ncb2324 21968997 PMC3462316

[28] Shao D, Oka S, Liu T, Zhai P, Ago T, Sciarretta S, et al A redox-dependent mechanism for regulation of AMPK activation by Thioredoxin1 during energy starvation. Cell metabolism. 2014;19(2):232–45. 10.1016/j.cmet.2013.12.013 24506865 PMC3937768

[29] Batinic-Haberle I, Benov L, Spasojevic I, Fridovich I. The ortho effect makes manganese(III) meso-tetrakis(N-methylpyridinium-2-yl)porphyrin a powerful and potentially useful superoxide dismutase mimic. The Journal of biological chemistry. 1998;273(38):24521–8. 9733746 10.1074/jbc.273.38.24521

[30] Day BJ, Fridovich I, Crapo JD. Manganic porphyrins possess catalase activity and protect endothelial cells against hydrogen peroxide-mediated injury. Arch Biochem Biophys. 1997;347(2):256–62. 10.1006/abbi.1997.0341 9367533

[31] Day BJ, Batinic-Haberle I, Crapo JD. Metalloporphyrins are potent inhibitors of lipid peroxidation. Free radical biology & medicine. 1999;26(5–6):730–6. 10218663 10.1016/s0891-5849(98)00261-5

[32] Batinic-Haberle I, Spasojevic I, Tse HM, Tovmasyan A, Rajic Z, St Clair DK, et al Design of Mn porphyrins for treating oxidative stress injuries and their redox-based regulation of cellular transcriptional activities. Amino acids. 2012;42(1):95–113. Epub 2010/05/18. 10.1007/s00726-010-0603-6 20473774 PMC3022969

[33] Batinic-Haberle I, Tovmasyan A, Spasojevic I. An educational overview of the chemistry, biochemistry and therapeutic aspects of Mn porphyrins—From superoxide dismutation to H2O2-driven pathways. Redox Biol. 2015;5:43–65. 10.1016/j.redox.2015.01.017 25827425 PMC4392060

[34] Padgett LE, Broniowska KA, Hansen PA, Corbett JA, Tse HM. The role of reactive oxygen species and proinflammatory cytokines in type 1 diabetes pathogenesis. Annals of the New York Academy of Sciences. 2013;1281:16–35. 10.1111/j.1749-6632.2012.06826.x 23323860 PMC3715103

[35] Delmastro MM, Styche AJ, Trucco MM, Workman CJ, Vignali DA, Piganelli JD. Modulation of redox balance leaves murine diabetogenic TH1 T cells "LAG-3-ing" behind. Diabetes. 2012;61(7):1760–8. Epub 2012/05/16. 10.2337/db11-1591 22586584 PMC3379669

[36] Piganelli JD, Flores SC, Cruz C, Koepp J, Batinic-Haberle I, Crapo J, et al A metalloporphyrin-based superoxide dismutase mimic inhibits adoptive transfer of autoimmune diabetes by a diabetogenic T-cell clone. Diabetes. 2002;51(2):347–55. Epub 2002/01/29. 11812741 10.2337/diabetes.51.2.347

[37] Sklavos MM, Tse HM, Piganelli JD. Redox modulation inhibits CD8 T cell effector function. Free radical biology & medicine. 2008;45(10):1477–86. Epub 2008/09/23. 18805480 10.1016/j.freeradbiomed.2008.08.023

[38] Tse HM, Milton MJ, Piganelli JD. Mechanistic analysis of the immunomodulatory effects of a catalytic antioxidant on antigen-presenting cells: implication for their use in targeting oxidation-reduction reactions in innate immunity. Free radical biology & medicine. 2004;36(2):233–47. Epub 2004/01/28. 14744635 10.1016/j.freeradbiomed.2003.10.029

[39] Delmastro-Greenwood MM, Votyakova T, Goetzman E, Marre ML, Previte DM, Tovmasyan A, et al Mn Porphyrin regulation of aerobic glycolysis: implications on the activation of diabetogenic immune cells. Antioxidants & redox signaling. 2013. Epub 2013/05/21. 10.1089/ars.2012.5167 PMC3931434 23682840

[40] Delong T, Wiles TA, Baker RL, Bradley B, Barbour G, Reisdorph R, et al Pathogenic CD4 T cells in type 1 diabetes recognize epitopes formed by peptide fusion. Science. 2016;351(6274):711–4. 10.1126/science.aad2791 26912858 PMC4884646

[41] Yoshida K, Martin T, Yamamoto K, Dobbs C, Munz C, Kamikawaji N, et al Evidence for shared recognition of a peptide ligand by a diverse panel of non-obese diabetic mice-derived, islet-specific, diabetogenic T cell clones. Int Immunol. 2002;14(12):1439–47. 12456592 10.1093/intimm/dxf106

[42] Nazarewicz RR, Bikineyeva A, Dikalov SI. Rapid and specific measurements of superoxide using fluorescence spectroscopy. J Biomol Screen. 2013;18(4):498–503. 10.1177/1087057112468765 23190737 PMC4210376

[43] Sukumar M, Liu J, Ji Y, Subramanian M, Crompton JG, Yu Z, et al Inhibiting glycolytic metabolism enhances CD8+ T cell memory and antitumor function. J Clin Invest. 2013;123(10):4479–88. 10.1172/JCI69589 24091329 PMC3784544

[44] Kuystermans D, Al-Rubeai M. cMyc increases cell number through uncoupling of cell division from cell size in CHO cells. BMC Biotechnol. 2009;9:76 10.1186/1472-6750-9-76 19735559 PMC2749834

[45] Nakajima EC, Laymon C, Oborski M, Hou W, Wang L, Grandis JR, et al Quantifying metabolic heterogeneity in head and neck tumors in real time: 2-DG uptake is highest in hypoxic tumor regions. PLoS One. 2014;9(8):e102452 10.1371/journal.pone.0102452 25127378 PMC4134191

[46] Good M, Siggers RH, Sodhi CP, Afrazi A, Alkhudari F, Egan CE, et al Amniotic fluid inhibits Toll-like receptor 4 signaling in the fetal and neonatal intestinal epithelium. Proc Natl Acad Sci U S A. 2012;109(28):11330–5. 10.1073/pnas.1200856109 22733781 PMC3396489

[47] Devadas S, Zaritskaya L, Rhee SG, Oberley L, Williams MS. Discrete generation of superoxide and hydrogen peroxide by T cell receptor stimulation: selective regulation of mitogen-activated protein kinase activation and fas ligand expression. The Journal of experimental medicine. 2002;195(1):59–70. Epub 2002/01/10. 10.1084/jem.20010659 11781366 PMC2196010

[48] Tse HM, Milton MJ, Schreiner S, Profozich JL, Trucco M, Piganelli JD. Disruption of innate-mediated proinflammatory cytokine and reactive oxygen species third signal leads to antigen-specific hyporesponsiveness. Journal of immunology. 2007;178(2):908–17. 10.4049/jimmunol.178.2.908 17202352

[49] Chiu J, Dawes IW. Redox control of cell proliferation. Trends in cell biology. 2012;22(11):592–601. Epub 2012/09/07. 10.1016/j.tcb.2012.08.002 22951073

[50] Kesarwani P, Murali AK, Al-Khami AA, Mehrotra S. Redox regulation of T-cell function: from molecular mechanisms to significance in human health and disease. Antioxidants & redox signaling. 2013;18(12):1497–534. Epub 2012/09/04. 22938635 10.1089/ars.2011.4073 PMC3603502

[51] Haskins K, McDuffie M. Acceleration of diabetes in young NOD mice with a CD4+ islet-specific T cell clone. Science. 1990;249(4975):1433–6. 2205920 10.1126/science.2205920

[52] Besson A, Dowdy SF, Roberts JM. CDK inhibitors: cell cycle regulators and beyond. Developmental cell. 2008;14(2):159–69. Epub 2008/02/13. 10.1016/j.devcel.2008.01.013 18267085

[53] Dong F, Agrawal D, Bagui T, Pledger WJ. Cyclin D3-associated kinase activity is regulated by p27kip1 in BALB/c 3T3 cells. Mol Biol Cell. 1998;9(8):2081–92. 9693368 10.1091/mbc.9.8.2081 PMC25461

[54] Pearce EL. Metabolism in T cell activation and differentiation. Current opinion in immunology. 2010;22(3):314–20. 10.1016/j.coi.2010.01.018 20189791 PMC4486663

[55] Chang CH, Curtis JD, Maggi LB Jr., Faubert B, Villarino AV, O'Sullivan D, et al Posttranscriptional control of T cell effector function by aerobic glycolysis. Cell. 2013;153(6):1239–51. 10.1016/j.cell.2013.05.016 23746840 PMC3804311

[56] Macintyre AN, Gerriets VA, Nichols AG, Michalek RD, Rudolph MC, Deoliveira D, et al The glucose transporter Glut1 is selectively essential for CD4 T cell activation and effector function. Cell metabolism. 2014;20(1):61–72. 10.1016/j.cmet.2014.05.004 24930970 PMC4079750

[57] Wieman HL, Wofford JA, Rathmell JC. Cytokine stimulation promotes glucose uptake via phosphatidylinositol-3 kinase/Akt regulation of Glut1 activity and trafficking. Mol Biol Cell. 2007;18(4):1437–46. 10.1091/mbc.E06-07-0593 17301289 PMC1838986

[58] Palmer CS, Ostrowski M, Gouillou M, Tsai L, Yu D, Zhou J, et al Increased glucose metabolic activity is associated with CD4+ T-cell activation and depletion during chronic HIV infection. AIDS. 2014;28(3):297–309. 10.1097/QAD.0000000000000128 24335483 PMC4293200

[59] Telang S, Clem BF, Klarer AC, Clem AL, Trent JO, Bucala R, et al Small molecule inhibition of 6-phosphofructo-2-kinase suppresses t cell activation. J Transl Med. 2012;10:95 10.1186/1479-5876-10-95 22591674 PMC3441391

[60] Yang Z, Fujii H, Mohan SV, Goronzy JJ, Weyand CM. Phosphofructokinase deficiency impairs ATP generation, autophagy, and redox balance in rheumatoid arthritis T cells. The Journal of experimental medicine. 2013;210(10):2119–34. 10.1084/jem.20130252 24043759 PMC3782046

[61] Delgoffe GM, Kole TP, Zheng Y, Zarek PE, Matthews KL, Xiao B, et al The mTOR kinase differentially regulates effector and regulatory T cell lineage commitment. Immunity. 2009;30(6):832–44. 10.1016/j.immuni.2009.04.014 19538929 PMC2768135

[62] Delgoffe GM, Pollizzi KN, Waickman AT, Heikamp E, Meyers DJ, Horton MR, et al The kinase mTOR regulates the differentiation of helper T cells through the selective activation of signaling by mTORC1 and mTORC2. Nature immunology. 2011;12(4):295–303. 10.1038/ni.2005 21358638 PMC3077821

[63] Fingar DC, Salama S, Tsou C, Harlow E, Blenis J. Mammalian cell size is controlled by mTOR and its downstream targets S6K1 and 4EBP1/eIF4E. Genes Dev. 2002;16(12):1472–87. 10.1101/gad.995802 12080086 PMC186342

[64] Kwon J, Shatynski KE, Chen H, Morand S, de Deken X, Miot F, et al The nonphagocytic NADPH oxidase Duox1 mediates a positive feedback loop during T cell receptor signaling. Sci Signal. 2010;3(133):ra59 10.1126/scisignal.2000976 20682913 PMC2941205

[65] Fracchia KM, Pai CY, Walsh CM. Modulation of T Cell Metabolism and Function through Calcium Signaling. Front Immunol. 2013;4:324 10.3389/fimmu.2013.00324 24133495 PMC3795426

[66] MacIver NJ, Blagih J, Saucillo DC, Tonelli L, Griss T, Rathmell JC, et al The liver kinase B1 is a central regulator of T cell development, activation, and metabolism. Journal of immunology. 2011;187(8):4187–98. 10.4049/jimmunol.1100367 PMC3206094 21930968

[67] Lienhardt C, Azzurri A, Amedei A, Fielding K, Sillah J, Sow OY, et al Active tuberculosis in Africa is associated with reduced Th1 and increased Th2 activity in vivo. Eur J Immunol. 2002;32(6):1605–13. 10.1002/1521-4141(200206)32:6<1605::AID-IMMU1605>3.0.CO;2-6 12115643

[68] Triebel F, Hacene K, Pichon MF. A soluble lymphocyte activation gene-3 (sLAG-3) protein as a prognostic factor in human breast cancer expressing estrogen or progesterone receptors. Cancer Lett. 2006;235(1):147–53. 10.1016/j.canlet.2005.04.015 15946792

[69] Boyman O, Sprent J. The role of interleukin-2 during homeostasis and activation of the immune system. Nature reviews Immunology. 2012;12(3):180–90. 10.1038/nri3156 22343569

[70] Triebel F, Jitsukawa S, Baixeras E, Roman-Roman S, Genevee C, Viegas-Pequignot E, et al LAG-3, a novel lymphocyte activation gene closely related to CD4. The Journal of experimental medicine. 1990;171(5):1393–405. 1692078 10.1084/jem.171.5.1393 PMC2187904

[71] Pollizzi KN, Sun IH, Patel CH, Lo YC, Oh MH, Waickman AT, et al Asymmetric inheritance of mTORC1 kinase activity during division dictates CD8(+) T cell differentiation. Nature immunology. 2016;17(6):704–11. 10.1038/ni.3438 27064374 PMC4873361

[72] Pollizzi KN, Waickman AT, Patel CH, Sun IH, Powell JD. Cellular size as a means of tracking mTOR activity and cell fate of CD4+ T cells upon antigen recognition. PLoS One. 2015;10(4):e0121710 10.1371/journal.pone.0121710 25849206 PMC4388710

[73] Dang CV. c-Myc target genes involved in cell growth, apoptosis, and metabolism. Molecular and cellular biology. 1999;19(1):1–11. 9858526 10.1128/mcb.19.1.1 PMC83860

[74] Bird JJ, Brown DR, Mullen AC, Moskowitz NH, Mahowald MA, Sider JR, et al Helper T cell differentiation is controlled by the cell cycle. Immunity. 1998;9(2):229–37. 9729043 10.1016/s1074-7613(00)80605-6

[75] Verbist KC, Guy CS, Milasta S, Liedmann S, Kaminski MM, Wang R, et al Metabolic maintenance of cell asymmetry following division in activated T lymphocytes. Nature. 2016;532(7599):389–93. 10.1038/nature17442 27064903 PMC4851250

[76] Chang JT, Palanivel VR, Kinjyo I, Schambach F, Intlekofer AM, Banerjee A, et al Asymmetric T lymphocyte division in the initiation of adaptive immune responses. Science. 2007;315(5819):1687–91. 10.1126/science.1139393 17332376

[77] Yang Z, Goronzy JJ, Weyand CM. The glycolytic enzyme PFKFB3/phosphofructokinase regulates autophagy. Autophagy. 2014;10(2):382–3. 10.4161/auto.27345 24351650 PMC5079104

